# Synthesis and biological activity evaluation of a novel pleuromutilin derivative 22-((2-methyl-1-(1H-pyrrole-2-carboxamido)propan-2-yl)thio)-deoxypleuromutilin

**DOI:** 10.3389/fphar.2025.1657973

**Published:** 2025-09-05

**Authors:** Zhun Li, Danqian Ma, Chang Liu, Zhe Qin, Lixia Bai, Wenbo Ge, Xiao Xu, Jianyong Li

**Affiliations:** Key Lab of New Animal Drug Project of Gansu Province, Key Lab of Veterinary Pharmaceutical Development of Ministry of Agriculture and Rural Affairs, Lanzhou Institute of Husbandry and Pharmaceutical Sciences of CAAS, Lanzhou, China

**Keywords:** pleuromutilin, PDP, antibacterial activity, toxicity, MRSA

## Abstract

**Objective and methods:**

Widespread antibiotic misuse has resulted in growing antimicrobial resistance, diminishing the clinical efficacy of existing antibiotics against resistant strains. Therefore, we designed and synthesized a novel pleuromutilin derivative **PDP**, and its antibacterial activities were evaluated *in vitro* and *in vivo*.

**Results:**

**PDP** exhibited potent antibacterial activity against Gram-positive bacteria (MRSA, MRSE, *Staphylococcus aureus, Streptococcus agalactiae* and *Staphylococcus dysgalactiae*), demonstrating a remarkably low MIC of 0.008 μg/mL, which was superior to both reference drugs tiamulin and valnemulin. Moreover, compared to tiamulin, it displayed a slower rate of resistance development. Molecular docking results demonstrate that **PDP** exhibits favorable binding to the peptidyl transferase center. The inhibition of bacterial protein synthesis by **PDP** was indirectly demonstrated through GFP expression inhibition assays. Derivative **PDP** exhibited extremely low cytotoxicity and had low oral acute toxicity, with an LD_50_ exceeding 2,000 mg/kg of body weight. When tested in a mouse model of systemic infection, **PDP** demonstrated superior efficacy to tiamulin and comparable activity to valnemulin. The bacterial carrier load indicated that **PDP** possessed significant efficacy in mitigating tissue damage resulting from MRSA infection in the lung, kidney, and liver.

**Conclusion:**

Consequently, **PDP** is a promising compound that may be useful for the development of therapeutic applications in the future.

## 1 Introduction

The growing severity of bacterial resistance and the emergence of “super bacteria” not only constitute widespread social and medical problems but also pose substantial challenges to global health and economic stability (Ding et al., 2022; Butler et al., 2017). The clinical efficacy of numerous antibacterial agents is being progressively compromised by the escalating prevalence of bacterial resistance (Astolfi et al., 2017). However, the pipeline for novel antibacterial agents has progressed at an alarmingly slow pace in recent decades (Devasahayam et al., 2010). There is an urgent need for novel therapeutic agents targeting drug-resistant bacterial pathogens (Brown and Wright, 2016; Mitcheltree et al., 2021; Durand-Reville et al., 2021). Modification of the parent scaffold of existing antibiotics is an effective means of developing new antibacterial drugs (Newman and Cragg, 2016).

**SCHEME 1:**
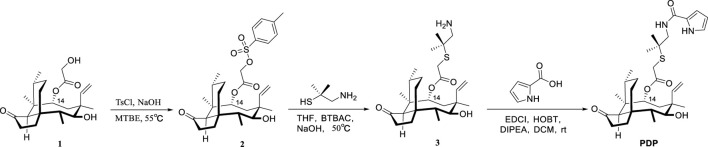
Synthesis of Pleuromutilin Derivative PDP.

**FIGURE 1:**
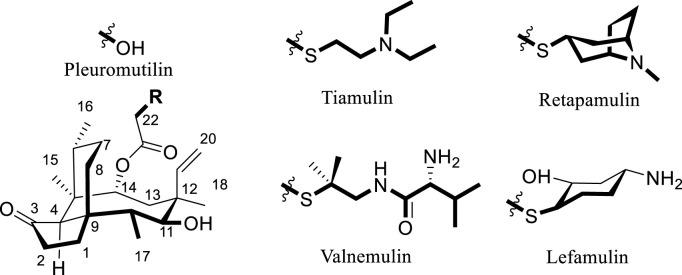
Approved drugs with pleuromutilin.

Semisynthetic modification of natural product scaffolds represents a pivotal approach for antibacterial drug discovery and development (George and Wright, 2025). The diterpenoid compound pleuromutilin was initially isolated in 1951 from basidiomycete fungi of the Pleurotus genus, specifically *P. mutilus* and *Pleurotus passeckerianus* (Kavanagh et al., 1951). Pleuromutilin possesses a distinctive tricyclic core structure containing eight continuous stereogenic centers (Figure 1). Pleuromutilin and its semisynthetic derivatives demonstrate potent antibacterial activity against Gram-positive and fastidious Gram-negative pathogens as well as against *mycoplasmas* and intracellular organisms, such as *Chlamydia* spp. and *Legionella pneumophila* (Li et al., 2024; Paukner and Riedl, 2017).

Pleuromutilin derivatives exert their antibacterial activity through selective inhibition of protein synthesis by binding to the peptidyl transferase center (PTC) located in domain V of the 50S ribosomal subunit (Qi et al., 2025). The tricyclic mutilin core of pleuromutilin derivatives specifically targets the ribosomal A-site, while their C-14 extended side chains occupy the adjacent P-site of the PTC, ultimately blocking mRNA translation (Liu Y. et al., 2024). Pleuromutilin and virginiamycin stand apart from other antibiotics by engaging both A- and P-sites simultaneously (Liu Y. et al., 2024; Yi et al., 2023). Owing to their distinctive mechanism of action, pleuromutilin derivatives exhibit minimal cross-resistance with conventional antibiotic classes (Yi et al., 2023).

Semisynthetic pleuromutilin derivatives have been modified either at the tricyclic core or C-14 side chain (Liu Y. et al., 2024; Llabani et al., 2019). Given the synthetic challenges and diminished bioactivity resulting from core structure modifications, most research efforts have focused on optimizing C-14 side chain derivatives (Liu et al., 2022). Notably, all clinically developed derivatives retain the intact tricyclic core structure (Yong et al., 2023). Tiamulin (1979) and Valnemulin (1999), two clinically important semisynthetic pleuromutilin derivatives (Figure 1), have been widely utilized as veterinary antibiotics (Liu K. et al., 2024). Approved by the U.S. in 2007, retapamulin was the first pleuromutilin antibiotic approved for human topical use (Luo et al., 2024). In 2019, the US FDA approved lefamulin, a systemic pleuromutilin antibiotic, for human use through both oral and intravenous administration routes (Eraikhuemen et al., 2021).

Nitrogen heterocycles serve as highly valuable structural components within the molecules of approved antibacterial drugs utilized for treating bacterial infections (Gomtsyan, 2012; Asif and Alghamdi, 2021). Pyrrole, a five - atom heterocycle, is present in a multitude of natural compounds that exhibit biological activities, among which antibacterial activity is notable. Numerous compounds are being develop based on the pyrrole heterocycle as new potential antibacterial drugs (Rusu et al., 2024).

Structure-activity relationship (SAR) studies demonstrate that incorporating a thioether moiety at the C-22 position of pleuromutilin significantly enhances its antibacterial efficacy. The thioether functional group, especially in conjunction with an optimized side chain, plays a pivotal role in determining the compound’s pharmacological profile (Paukner and Riedl, 2017; Liu Y. et al., 2024; Goethe et al., 2019; Wang et al., 2022). In this study, derivative **PDP** were synthesized by sequentially introducing dimethylcysteamine and pyrrole groups to the pleuromutilin core, aiming to enhance activity against drug-resistant bacterial infections.

## 2 Results and discussion

### 2.1 Chemistry

22-((2-methyl-1-(1H-pyrrole-2-carboxamido)propan-2-yl)thio)-deoxy pleuromutilin (compound **PDP**) was prepared following the previously described methods (Li et al., 2024; Liu K. et al., 2024; Yi et al., 2025; zhang et al., 2018). With the assistance of dimethylcysteamine, the target pleuromutilin derivative **PDP** was synthesized by incorporating a pyrrole group into pleuromutilin. Pleuromutilin was first converted to intermediate **2**, which was then reacted with dimethylcysteamine hydrochloride to afford intermediate **3** (zhang et al., 2018; Shang et al., 2014). In the current study, intermediate **3** was synthesized using a 20% (m/v) aqueous NaOH solution under rigorously controlled reaction conditions, including optimized temperature and time parameters (Scheme 1).

Pleuromutilin derivative **PDP** was synthesized by reacting intermediate **3** with prrole-2-carboxylic acid in the presence of either EDCI, HOBT, and DIPEA at room temperature. The yield of pleuromutilin derivative **PDP** was 53.4%. The purity of the target compounds was determined using high - performance liquid chromatography with an eluent consisting of acetonitrile in water. The results showed that the purity of the target compounds exceeded 95.0%. The synthesized derivative **PDP** was characterized using proton nuclear magnetic resonance spectroscopy (^1^H NMR), carbon - 13 nuclear magnetic resonance spectroscopy (^13^C NMR), and high - resolution mass spectra (HR MS) analytical techniques (Supplementary Data Sheet 1).

### 2.2 Antibacterial activity

The *in vitro* antibacterial activity of **PDP** was assessed against nine pathogenic bacterial strains. These strains included *Staphylococcus aureus* (ATCC 29213), *Methicillin-resistant Staphylococcus epidermidis* (MRSE, ATCC 51625), *Methicillin-resistant S. aureus* (MRSA, ATCC 43300), *Escherichia coli* (ATCC 25922), including clinical isolates of *Streptococcus agalactiae* and three distinct *Staphylococcus dysgalactiae* strains (designated as *S. dysgalactiae*-1, -2, and -3). Infections caused by these pathogens pose life-threatening risks and constitute a major public health concern. Of particular significance, MRSA has emerged as a predominant causative agent of hospital-acquired infections (Hammad et al., 2019). *Streptococcus* is capable of infecting a broad spectrum of both animals and humans (Cai et al., 2023; Torres et al., 2023). To further characterize its anti-MRSA activity, derivative **PDP** was evaluated against a panel of 48 clinical MRSA isolates *in vitro*. Forty-eight clinical MRSA isolates associated with dairy farms were collected from four Chinese provinces (Gansu, Shanghai, Sichuan, and Guizhou) (Zhou et al., 2023). These isolates were provided by the Lanzhou Institute of Husbandry and Pharmaceutical Sciences of the Chinese Academy of Agricultural Sciences.

Tiamulin and valnemulin were employed as reference compounds. Preliminary testing of the derivative **PDP** against nine bacterial strains demonstrated potent activity against Gram-positive bacteria, outperforming the control drugs tiamulin and valnemulin. The MIC of derivative **PDP** against tested Gram-positive bacteria is 0.008–0.016 μg/mL. However, derivative **PDP** exhibited limited activity against *E. coli*, demonstrating a minimum inhibitory concentration (MIC) of ≥16 μg/mL (Table 1).

**TABLE 1:** *In vitro* antibacterial activity of derivative PDP.

Cpd.	MICs (µg/mL)							
	MRSA	MRSE	S.aur^1^	S.ag	S.dys^1^	S.dys^2^	S.dys^3^	Ec
PDP	0.008	0.016	0.016	0.008	0.008	0.008	0.008	>16
Tiamulin	0.25	0.25	0.5	0.5	0.25	0.25	0.25	>16
Valnemulin	0.031	0.031	0.031	0.031	0.031	0.016	0.016	16

S.aur^1^ (*S.aureus*-29213); S.ag (*S.agalactiae*); S.dys^1^ (*S.dysgalactiae*-1); S.dys^2^ (*S.dysgalactiae*-2); S.dys^3^ (*S.dysgalactiae*-3); Ec (*Escherichia coli*-25922).

The MIC ranges of **PDP**, tiamulin and valnemulin against MRSA clinical strains were 0.008–0.063, 0.125–1, and 0.031–0.25 μg/mL, respectively. The derivative **PDP** demonstrated significantly superior activity against MRSA clinical isolates compared to the control drugs tiamulin and valnemulin (Table 2).

**TABLE 2:** *In vitro* antibacterial activity of derivative PDP.

Strains	MICs (µg/mL)			Strains	MICs (µg/mL)		
	PDP	Tiamulin	Valnemulin		PDP	Tiamulin	Valnemulin
1	0.063	1	0.031	25	0.031	0.5	0.063
2	0.031	0.5	0.063	26	0.016	0.5	0.063
3	0.031	0.5	0.25	27	0.008	0.5	0.063
4	0.063	0.5	0.25	28	0.031	1	0.063
5	0.008	0.5	0.063	29	0.008	1	0.063
6	0.016	0.5	0.125	30	0.016	0.5	0.063
7	0.008	0.5	0.063	31	0.008	0.5	0.063
8	0.008	0.25	0.063	32	0.008	1	0.063
9	0.008	1	0.063	33	0.008	1	0.063
10	0.031	0.5	0.063	34	0.031	0.5	0.063
11	0.031	0.5	0.063	35	0.031	0.5	0.063
12	0.016	0.5	0.031	36	0.008	0.25	0.063
13	0.008	0.25	0.063	37	0.008	0.5	0.063
14	0.063	0.5	0.125	38	0.016	0.5	0.063
15	0.031	0.5	0.063	39	0.016	0.25	0.125
16	0.016	0.5	0.063	40	0.016	1	0.063
17	0.031	0.5	0.063	41	0.016	0.5	0.063
18	0.031	0.125	0.063	42	0.016	0.5	0.063
19	0.063	0.5	0.063	43	0.016	1	0.063
20	0.031	0.5	0.125	44	0.016	0.5	0.063
21	0.031	0.5	0.125	45	0.031	1	0.25
22	0.031	0.5	0.25	46	0.016	1	0.063
23	0.031	0.5	0.063	47	0.016	0.5	0.063
24	0.031	0.25	0.063	48	0.016	0.5	0.125

### 2.3 Time-kill kinetic study

**FIGURE 2:**
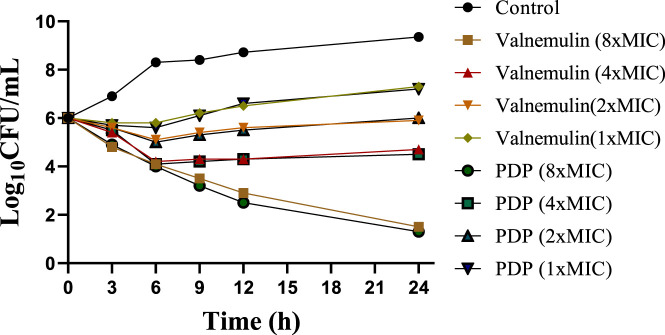
Time-kill curves of valnemulin and **PDP** against MRSA ATCC 43300.

To evaluate derivative **PDP**, we performed time-kill assays against MRSA at 1, 2, 4, 8 × MIC concentrations, using valnemulin as the reference drug. Both compounds showed concentration-dependent bactericidal activity (Figure 2). At concentrations of 1 × MIC and 2 × MIC, derivative **PDP** was observed to slow bacterial propagation. Specifically, in the 1×MIC treatment group, the bacterial concentration at 24 h remained higher than the initial inoculation concentration but was significantly lower than the bacterial concentration in the untreated control group. In the 2 × MIC treatment group, the bacterial concentration at 24 h was comparable to the initial inoculation concentration. At a concentration of 4 × MIC, derivative **PDP** exhibited a sterilizing effect, with the bacterial concentration at 24 h being lower than the initial inoculation concentration. When administered at 8×MIC, derivative **PDP** was able to almost completely eliminate MRSA within 24 h.

### 2.4 Resistance study

**FIGURE 3:**
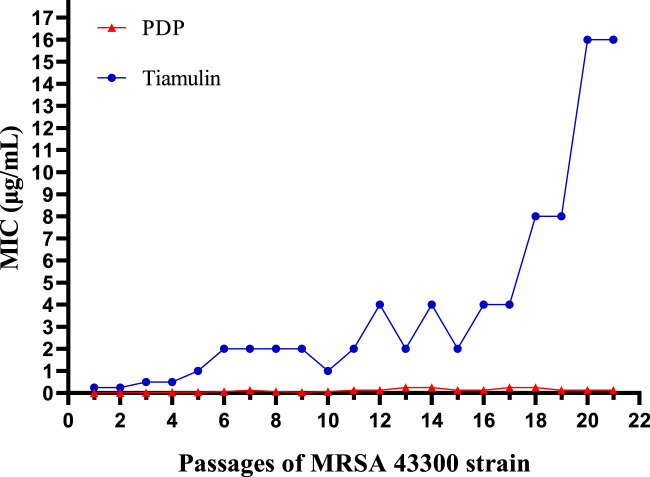
Development of MRSA ATCC 43300 resistance after repetitive treatment with derivative **PDP** or tiamulin for 21 passages.

To further assess the risk of microbial resistance development to derivative **PDP** during long-term use (Li et al., 2021), an induced drug resistance assay was conducted using MRSA 43300 (Figure 3). Tiamulin exhibited progressive resistance development, with MIC values increasing from baseline to 2 μg/mL (8-fold) by day 6, 4 μg/mL (16-fold) by day 12, and reaching 16 μg/mL (64-fold increase) by day 20. In contrast, derivative **PDP** showed only minimal resistance emergence, with a final MIC of 0.125 μg/mL. The observed resistance progression indicates a significantly slower development of resistance to **PDP** derivative compared with tiamulin.

### 2.5 Molecular docking study and inhibiting bacterial protein expression

**FIGURE 4:**
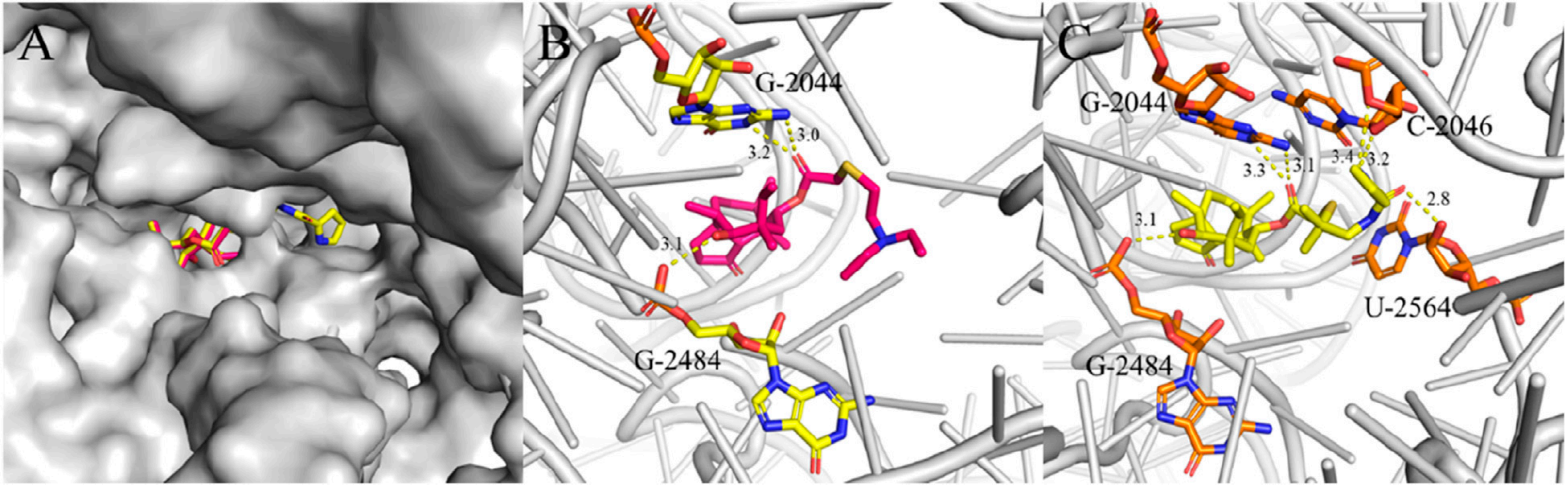
Interactions between ligands with PTC residues. Superimposed poses of selected tiamulin (red) and **PDP** (yellow) in the PTC of the 50S ribosomal **(A)**. Interactions between tiamulin with residues **(B)** and **PDP** with residues **(C)**.

Derivative **PDP** exhibited excellent *in vitro* antibacterial activity. To further investigate its mechanism of action, we explored the binding mode of **PDP** to the 50S ribosomal subunit (PDB ID: 1XBP). As a reference, tiamulin was docked into the same target using Smina, and the resulting root mean square deviation (RMSD) value was <2.0 Å (Figure 4; Trott and Olson, 2010; Quiroga and Villarreal, 2016).

The structural diagram of the ligand-receptor complex demonstrates that both tiamulin and **PDP** bind exclusively to ribosomal RNA. In the structural representation, the 50S ribosomal subunit core is shown in gray cartoon format, with ligands and their hydrogen-bonding residues rendered as stick models. Hydrogen bonds were formed between the hydroxyl groups located on the eight-membered rings of the docked compounds and the G-2484 residues, and these interactions were identified as key binding forces in the complex (Figures 4B,C). The ester carbonyl groups in the side chains of tiamulin and **PDP** form hydrogen bonds with the G-2044 residue (Figures 4B,C). Additionally, the acylamide carbonyl and pyrrole group on the **PDP** side chain form hydrogen bonds with residue U-2564 and C-2046, respectively (Figure 4C). When flexibly docked into the PTC, derivative **PDP** exhibited a higher binding affinity (ΔGb = −10.7 kcal/mol) compared to tiamulin (ΔGb = −9.0 kcal/mol). This difference in binding affinity is consistent with their respective antibacterial activities.

**FIGURE 5:**
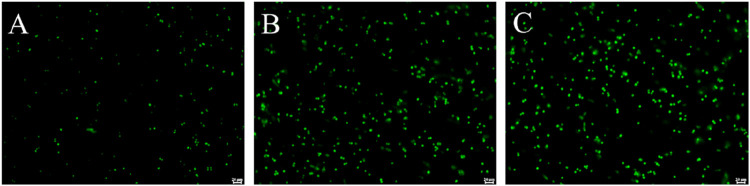
Derivative **PDP** inhibited GFP expression in *Staphylococcus aureus* ATCC 43000. **(A)** 0.25 μg/mL PDP, **(B)** 2 μg/mL Tiamulin **(C)** Control group. Scale bar: 20 μm.

Given the strong antibacterial activity of **PDP**, we proceeded to assess its impact on protein synthesis using modified *Staphylococcus aureus* strains that ectopically express a green fluorescent protein (GFP) (Liu et al., 2023). The objective was to observe the reduction in green fluorescence intensity, which indicates hindered protein synthesis. Following a 4-h co-incubation with the GFP strains, it was evident that 0.25 μg/mL **PDP** can effectively suppress bacterial GFP expression compared with the control (Figure 5). The average fluorescence intensity results showed that compared to the control group, the fluorescence intensity decreased by 30.29% in the tiamulin group and by 79.99% in the **PDP** group. Similar to our earlier findings with pleuromutilin derivatives, this result was obtained using the BCA method (Qi et al., 2025).

### 2.6 Safety evaluation

**FIGURE 6:**
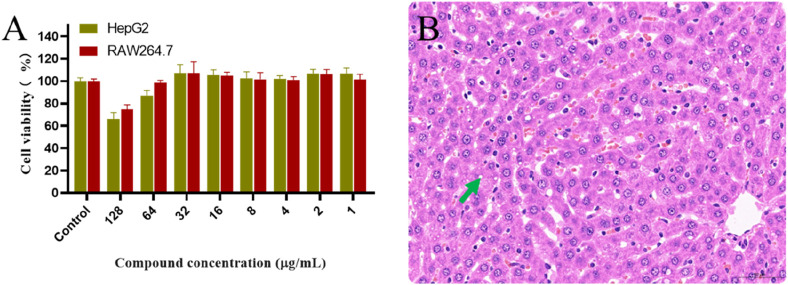
Toxological effects of **PDP**. **(A)** Effect of derivative **PDP** on proliferation of HepG2 and RAW264.7 cells, **(B)** The hepatic pathology revealed mild steatosis of hepatocytes (green arrow).

Cytotoxicity assays were performed to evaluate the toxicity of derivative **PDP** against the mammalian cell lines HepG2 and RAW264.7 using the CCK-8 assay (Figure 6A). At a concentration of 128 μg/mL, derivative **PDP** induced viabilities of 65.88% in HepG2 cells and 74.66% in RAW264.7 cells, respectively. The half-maximal inhibitory concentration (IC_50_) values of derivative **PDP** were ≥128 μg/mL for both cell types. These results indicated that derivative **PDP** exhibited low cytotoxicity toward mammalian cells, demonstrating satisfactory biocompatibility.

In the acute oral toxicity study conducted per OECD Guideline 423, no mortality or clinical abnormalities were observed in rats during the 24-h post-administration period or subsequent 7-day observation. All animals maintained normal physiological parameters including food intake, grooming behavior, neurological status, and excretory functions. Histopathological examination showed no significant tissue alterations in cardiac, splenic, pulmonary, or renal specimens from either control or treated groups. The 2000 mg/kg **PDP**-treated group exhibited only mild hepatic steatosis (Figure 6B), with no other drug-related pathology. Based on these findings, the LD_50_ of derivative **PDP** was determined to exceed 2000 mg/kg body weight.

### 2.7 *In vivo* efficacy in mice model of systemic infection

**FIGURE 7:**
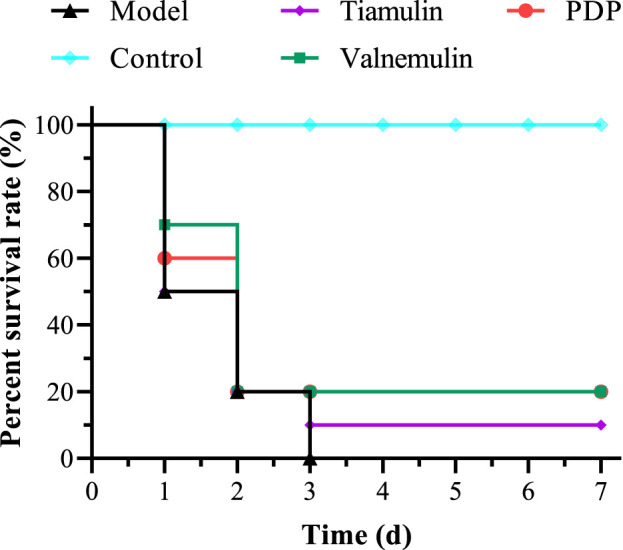
Survival rates of **PDP**, tiamulin and valnemulin in mice systemic infection models.

In the MRSA systemic infection model, mice received tail vein injections of **PDP** derivative, tiamulin, or valnemulin (20 mg/kg). Treatment with **PDP** (20 mg/kg) resulted in a 20% survival rate, demonstrating superior efficacy to tiamulin (10% survival) and equivalent activity to valnemulin (20% survival) at the same dose (Figure 7). Compared with the model, **PDP** significantly delayed death and improved survival rate.

**FIGURE 8:**
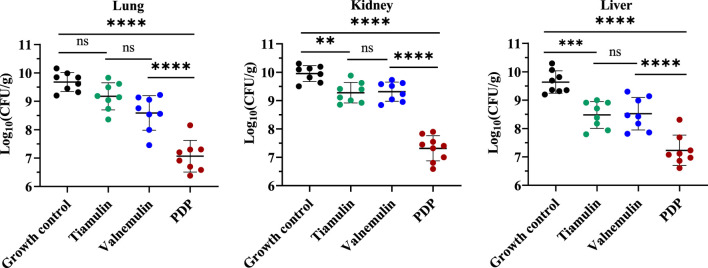
Efficacy of **PDP**, tiamulin and valnemulin in mice systemic infection model. Bacterial load of MRSA in lung, kidney and liver of infected mice. Values are means and standard errors. **p < 0.01, ****p < 0.0001.

We subsequently quantified bacterial burdens in the lungs, liver, and kidneys of mice following 7-day treatment (Figure 8). At equivalent doses, derivative **PDP** exhibited a significant reduction in the mean bacterial load in the lungs (2.109 and 1.522 Log_10_ CFU), kidneys (1.959 and 1.996 Log_10_ CFU), and liver (1.245 and 1.289 Log_10_ CFU) when compared to tiamulin and valnemulin, respectively (p < 0.0001). These results demonstrate that the **PDP** derivative effectively attenuates MRSA-induced pathological damage across pulmonary, renal and hepatic tissues.

**FIGURE 9:**
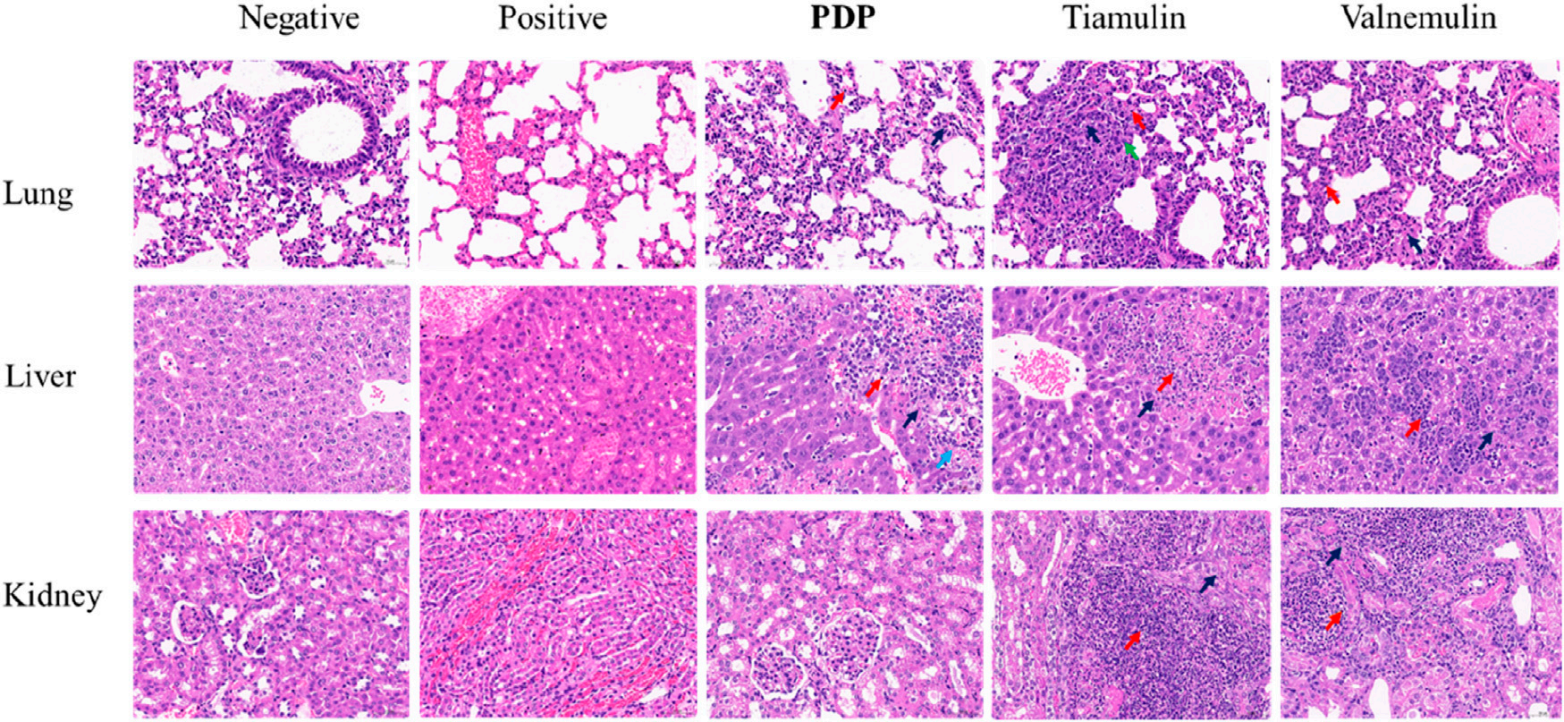
Representative H&E-stained sections of the main organs after various treatments (20 mg/kg of compound **PDP** and tiamulin) pathological section at ×400 magnification with a scale bar of 50 μm. Red arrows: inflammatory, black arrows: necrosis, green arrow: proliferation, blue arrow: hemorrhage.

Histopathological examination revealed alveolar cell deformation and necrosis with pronounced inflammatory infiltration in pulmonary tissues from tiamulin- and valnemulin-treated groups. Renal sections similarly exhibited marked tubular degeneration and necrosis accompanied by inflammatory cell accumulation. Compared with the control drug treatment group, **PDP** had milder lung tissue lesions and more severe liver lesions. In addition, no significant pathological changes were observed in the tissue of **PDP** kidneys (Figure 9).

### 2.8 In silico ADMET and druglikeness prediction

In the early stages of compound research and development, computer software can simulate and predict the ADMET properties of drugs. This capability allows researchers to forecast compound characteristics based on their chemical structures and assess the feasibility of potential drug candidates. For the present study, the pharmacokinetic properties of derivative **PDP** were predicted using the Swiss ADME platform. Table 3 summarizes the key pharmacokinetic profiles. The predicted lipophilicity values (log P (o/w)) for derivative **PDP**, tiamulin, and valnemulin were 4.52, 4.69, and 4.22, respectively, suggesting limited water solubility for all three compounds. Pharmacokinetic predictions indicated that tiamulin displayed high gastrointestinal absorption, whereas the absorption of derivative **PDP** and valnemulin was relatively lower. None of the three compounds can cross the blood-brain barrier (BBB); but valnemulin and PDP are substrates of P-glycoproteins. Derivative **PDP** may exert an inhibitory effect on CYP3A4 but shows no inhibition of CYP1A2, CYP2C19, CYP2C9, or CYP2D6. This finding is consistent with the characteristics of tiamulin and valnemulin. The skin permeability coefficients (Kp) for derivative **PDP**, tiamulin, and valnemulin were determined to be −5.74, −5.30, and −6.0 cm/s, respectively. Additionally, all three compounds achieved identical bioavailability scores.

**TABLE 3:** Predicted pharmacokinetic propertied for tiamulin, valnemulin and **PDP** by Swiss ADME.

Compounds	Tiamulin	Valnemulin	PDP
MW(g/mol)	493.74	564.82	558.77
GI absorption	High	Low	Low
BBB permeant	No	No	No
P-gp substrate	No	Yes	Yes
CYP1A2 inhibitor	No	No	No
CYP2C19 inhibitor	No	No	No
CYP2C9 inhibitor	No	No	No
CYP2D6 inhibitor	No	No	No
CYP3A4 inhibitor	Yes	Yes	Yes
log Kp (cm/s)	−5.30	−6.00	−5.74
log P (o/w)	4.69	4.22	4.52
Bioavailability Score	0.55	0.55	0.55

## 3 Conclusion

Pleuromutilin was used as the starting material, and multiple chemical reactions, including sulfonation, nucleophilic substitution, and amidation, were performed to obtain compound **PDP**. Preliminary testing of the derivative against nine bacterial strains demonstrated excellent activity against Gram-positive bacteria, outperforming the control drugs tiamulin and valnemulin. Compared to tiamulin, it displayed a slower rate of resistance development. Molecular docking and protein synthesis results showed that **PDP** was superior to tiamulin. In addition, **PDP** demonstrated good safety and therapeutic effects. Given these findings, Derivative **PDP** emerges as a highly promising compound, holding great potential for the future development of therapeutic applications.

## 4 Materials and methods

### 4.1 Chemistry

All reagents and solvents were purchased from Macklin Biochemical Co., Ltd., (Shanghai, China) and Sinopharm Chemical Reagent Limited Corporation. Reaction progress was monitored by thin-layer chromatography (TLC) using silica gel-coated plates, with visualization achieved using either ultraviolet (UV) light or phosphomolybdic acid staining. All pleuromutilin derivatives were purified via column chromatography using 200–300 mesh silica gel. Proton nuclear magnetic resonance (^1^H NMR) and carbon-13 nuclear magnetic resonance (^13^C NMR) spectra were recorded on Bruker spectrometers operating at 400 MHz and 101 MHz, respectively. High-resolution mass spectra (HRMS) were acquired using an Agilent Technologies 6530 Precision Mass Q-TOF LC/MS system.

### 4.2 Synthesis of 14-O-(((1-amino-2-methylpropan-2-yl)thio)acetyl)mutilin (3)

A mixture of pleuromutilin (5.0 g, 13.2 mmol) and *p*-toluenesulfonyl chloride (2.8 g, 14.5 mmol) in methyl tert-butyl ether (13.2 mL) was treated with the dropwise addition of 10 M sodium hydroxide solution (2.2 mL). The reaction mixture was stirred vigorously at 55 °C for 1 h. After the reaction, compound 2 was isolated by filtration, washed sequentially with water and methyl tert-butyl ether, and then dried in a desiccator. The product was obtained as a white solid (6.5 g, 93% yield).

A mixture of compound **2** (11.1 g, 20.8 mmol) and dimethylcystamine hydrochloride (9.0 g, 41.6 mmol) in tetrahydrofuran (80 mL) was treated with the dropwise addition of benzyltributylammonium chloride (0.65 g, 2.1 mmol) and 20% sodium hydroxide solution (15 mL). The reaction mixture was stirred at 50 °C for 4 h. After completion of the reaction, the products were extracted with dichloromethane. The organic extract was dried over anhydrous sodium sulfate, concentrated under reduced pressure, and subsequently purified by column chromatography using a solvent system consisting of petroleum ether, ethyl acetate, and diethylamine. Yellow powder, yield: 88.0%. ^1^H NMR (400 MHz, CDCl_3_) δ 6.47 (dd, *J* = 17.4, 11.0 Hz, 1H), 5.74 (d, *J* = 8.5 Hz, 1H), 5.33 (dd, *J* = 11.0, 1.5 Hz, 1H), 5.19 (dd, *J* = 17.4, 1.6 Hz, 1H), 3.34 (d, *J* = 6.5 Hz, 1H), 3.12 (d, *J* = 1.6 Hz, 2H), 2.59 (s, 2H), 2.39–2.26 (m, 1H), 2.27–2.15 (m, 2H), 2.13–2.01 (m, 2H), 1.76 (dd, *J* = 14.4, 3.1 Hz, 1H), 1.68–1.62 (m, 2H), 1.57–1.48 (m, 4H), 1.45 (s, 3H), 1.43–1.37 (m, 1H), 1.31 (d, *J* = 16.1 Hz, 1H), 1.23 (s, 6H), 1.16 (s, 3H), 1.10 (dd, *J* = 13.9, 4.4 Hz, 1H), 0.86 (d, *J* = 7.0 Hz, 3H), 0.72 (d, *J* = 6.9 Hz, 3H). ^13^C NMR (101 MHz, CDCl_3_) δ 217.30, 169.68, 139.29, 117.49, 74.86, 69.58, 58.47, 51.93, 48.80, 45.72, 44.99, 44.18, 42.05, 37.04, 36.27, 34.73, 31.51, 30.71, 29.95, 27.11, 26.59, 26.49, 25.11, 17.12, 15.19, 11.78. HRMS (ESI+): calcd for C_26_H_43_NO_4_S [M + H]^+^, 466.2986; found, 466.2970.

### 4.3 Synthesis of pleuromutilin derivative 22-((2-methyl-1-(1H-pyrrole-2-carbox amido)propan-2-yl)thio)-deoxypleuromutilin

Pyrrole-2-carboxylic acid (1.06 mmol) and compound 3 (0.49 g, 1.06 mmol) were dissolved in anhydrous dichloromethane (10 mL), followed by sequential addition of EDCI (0.24 g, 1.27 mmol), HOBt (0.17 g, 1.27 mmol), and N, N-diisopropylethylamine (DIPEA, 0.37 mL, 2.12 mmol). The reaction mixture was stirred at room temperature for 12–18 h (monitored by TLC). Sodium bicarbonate solution was added to the reaction mixture after complete reaction. Then, the products were extracted with dichloromethane, dried over anhydrous sodium sulfate, concentrated, and purified by column chromatography (Petroleum ether, ethyl acetate and diethylamine). White solid, yield: 53.4%. ^1^H NMR (400 MHz, CDCl_3_) δ 9.79 (s, 1H), 6.97–6.90 (m, 1H), 6.81–6.73 (m, 1H), 6.46 (dd, *J* = 17.4, 11.0 Hz, 1H), 6.28–6.22 (m, 1H), 5.75 (d, *J* = 8.5 Hz, 1H), 5.25–5.07 (m, 1H), 3.46–3.33 (m, 2H), 3.29–3.11 (m, 3H), 2.36–2.29 (m, 1H), 2.28–2.16 (m, 2H), 2.14–2.06 (m, 2H), 1.77 (dd, *J* = 14.5, 3.1 Hz, 1H), 1.71–1.50 (m, 4H), 1.45 (s, 3H), 1.42–1.31 (m, 2H), 1.29 (d, *J* = 7.2 Hz, 6H), 1.19–1.07 (m, 4H), 0.87 (d, *J* = 7.0 Hz, 3H), 0.72 (d, *J* = 7.0 Hz, 3H). ^13^C NMR (101 MHz, CDCl_3_) δ 217.19, 170.79, 161.37, 138.97, 126.34, 121.66, 117.62, 109.94, 109.57, 74.81, 70.43, 58.34, 47.98, 47.57, 45.68, 45.15, 44.24, 42.04, 36.93, 36.21, 34.70, 31.75, 30.65, 27.13, 26.63, 26.59, 26.53, 25.08, 17.21, 15.13, 11.81. HRMS (ESI+): calcd for C_31_H_46_N_2_O_5_S [M + H]^+^, 559.3200; found, 559.3199.

### 4.4 *In vitro* antibacterial activity

#### 4.4.1 MIC test

Minimum inhibitory concentration (MIC) experiments were conducted in accordance with the guidelines established by the Clinical and Laboratory Standards Institute (CLSI) (Li et al., 2025). Stock solutions of all compounds were prepared by dissolving them in DMSO at a concentration of 12.8 mg/mL and stored at −80 °C. Tiamulin was used as the reference drug. Prior to the experiment, the stock solutions were diluted to the required concentrations using Mueller-Hinton Broth (MHB). The final concentration of the bacterial inoculum in MHB was adjusted to approximately 10^5^ CFU/mL. In sterile 96-well plates, 100 μL of the bacterial suspension was added to 100 μL aliquots of the diluted compounds, followed by incubation at 37 °C for 24 h. The MIC of each compound was determined based on the observation of broth clarification (indicating inhibited bacterial growth).

#### 4.4.2 Bactericidal time-kill kinetics

MRSA was cultured in 6 mL of MHB at 37 °C for 12 h, after which the bacterial culture was diluted in MHB to a concentration of approximately 10^6^ CFU/mL. Compound PDP was then added to the prepared bacterial suspension at final concentrations of 1×, 2×, 4×, and 8× the minimum inhibitory concentration (MIC). At various time points (0, 3, 6, 9, 12, and 24 h), 100 μL aliquots of the bacterial suspension were collected, serially diluted 10-fold in sterile 0.9% saline, and inoculated onto sterile Mueller-Hinton agar plates. These plates were incubated at 37 °C for 24 h. The entire procedure was performed in triplicate, and the resulting colonies were counted, with results reported as log_10_ (CFU/mL).

#### 4.4.3 Drug resistance induction test

The development of drug resistance has emerged as a critical challenge in antibiotic research (Li et al., 2021). Consequently, evaluating the potential for bacterial resistance to these biocides is of utmost importance. We conducted an induced resistance assay on compound **PDP**, using tiamulin as the control drug. Specifically, MRSA was cultured overnight in 5 mL of MHB at a sub-minimum inhibitory concentration (sub-MIC). Over 21 consecutive passages, the resistance of MRSA to compound **PDP** and tiamulin was assessed by monitoring changes in the bacterial minimum inhibitory concentration (MIC). Resistance was defined as a greater than four-fold increase relative to the initial MIC.

### 4.5 Molecular docking study

Molecular docking simulations were performed using Smina software (Trott and Olson, 2010; Quiroga and Villarreal, 2016). Ligand energy minimization was carried out using Chem3D. For the X-ray crystal structure (PDB ID: 1XBP), ligands, all water molecules, and metal ions were first removed. The binding pocket was defined with coordinates (x: 52.8, y: 122.3, z: 113.8) and a dimension of 40 × 50 × 40 Å. To validate the docking protocol, the co-crystal ligand tiamulin was redocked, and its conformation was compared with the original structure in the crystal. Finally, all docking conformations were visualized using PyMOL software.

### 4.6 GFP inhibition assay

Plasmids harboring erythromycin resistance genes and labeled with green fluorescent protein (GFP) were introduced into *Staphylococcus aureus* via electro-transformation, as described previously (Liu et al., 2023). Following transformation, erythromycin-based screening was performed to isolate successfully transformed bacterial colonies. The selected *S. aureus* strain was cultured until it reached the mid-logarithmic growth phase. Bacterial cells were then harvested by centrifugation, resuspended in phosphate-buffered saline (PBS), and co-incubated with compound **PDP** and tiamulin at final concentrations of 0.25 μg/mL and 2 μg/mL, respectively. This co-incubation was conducted at 37 °C for 4 h. Afterward, the cells were recollected, resuspended in PBS once more, and their fluorescence was observed using fluorescence microscopy.

### 4.7 Safety evaluation

#### 4.7.1 Cytotoxicity assay

Cytotoxicity was evaluated using the Cell Counting Kit-8 (CCK-8) assay, as previously described (Li et al., 2022). HepG2 and RAW264.7 cells were utilized in this experiment. Specifically, 5.0 × 10^3^ cells were seeded into 96-well plates and incubated at 37 °C for 24 h. After removing the original culture medium, the cells were replenished with fresh medium containing varying concentrations of compound **PDP**. For the negative control group, cells were treated with culture medium supplemented with a corresponding concentration of DMSO. Following 24 h of treatment, the culture medium was aspirated, and the cells were washed twice with PBS. Subsequently, new medium containing 5% CCK-8 was added to each well, and the plate was incubated at 37 °C for 2 h. Finally, the absorbance was measured at 450 nm using a microplate reader.

#### 4.7.2 Acute oral toxicity assay

Acute oral toxicity tests were performed in accordance with the guidelines specified in the OECD 423 bulletin. Healthy female Sprague-Dawley (SD) rats, aged 8 weeks and weighing 200 ± 10 g, were chosen for the study. Prior to the initiation of the experiment, the rats were allowed a 7-day acclimation period. Prior to the experiment, the rats were fasted and deprived of water for 12 h. For the first round of gavage toxicity testing, three rats were selected and administered a dose of 2,000 mg/kg. Observations were conducted at 4 h, 12 h, and 1-day post-administration, with monitoring continuing until the seventh day. During this period, no deaths or other abnormal phenomena were observed. Consequently, the remaining seven rats were subjected to gavage at the same dose. After 7 days of gavage, the rats were weighed and subsequently euthanized using ether. The heart, liver, spleen, lungs, and kidneys were fixed in 4% tissue fixative, followed by paraffin embedding. The embedded tissues were sectioned at a thickness of 4 μm, stained with hematoxylin and eosin (H&E), and subsequently examined under a light microscope.

### 4.8 *In Vivo* efficacy in a mouse model of systemic infection

Six-week-old SPF Kunming mice (weighing 25–30 g) were obtained from the Lanzhou Institute of Veterinary Medicine, Chinese Academy of Agricultural Sciences.

The mice were randomly divided into five groups (n = 10 per group) with balanced gender distribution. Control drugs (tiamulin and valnemulin) and compound **PDP** were dissolved in a vehicle solution containing 5% DMSO, 10% Tween 80, and 85% saline, followed by sterile filtration. Four days prior to inoculation, all mice received an intraperitoneal injection of cyclophosphamide at a dosage of 150 mg/kg. Subsequently, 1 day before inoculation, they were administered another intraperitoneal injection of cyclophosphamide at a reduced dosage of 100 mg/kg. Next, neutropenic mice were given an intraperitoneal injection of 0.56 mL MRSA inoculum with a concentration of 10^8^ CFU/mL. Half an hour later, those mice that had received the MRSA inoculum were injected with the compound at a dose of 20 mg/mL via the tail vein. The daily mortality of the mice was monitored over a 7-day period. After this period, the mice were anesthetized with an excess of carbon dioxide, and their lungs, livers, and kidneys were harvested.

### 4.9 Predicted pharmacokinetic properties

Swiss ADME was employed to analyze the pharmacokinetic parameters of tiamulin, valnemulin, and **PDP**.

### 4.10 Ethics statement

All animal experiments strictly adhered to the Animal Ethics Procedures and Guidelines of the People’s Republic of China. This research procedure received approval from the Animal Administration and Ethics Committee of Lanzhou Institute of Husbandry and Pharmaceutical Sciences of CAAS (Permit NO: SYXK-2023-006).

### 4.11 Statistical analysis

Statistical analyses were performed using IBM SPSS 24.0. One-way ANOVA with Dunnett’s *post hoc* testing was employed, with significance levels defined as p < 0.05 (significant) and p < 0.01 (highly significant).

## Data Availability

The original contributions presented in the study are included in the article/Supplementary Material, further inquiries can be directed to the corresponding author.
