# Supplementary material for: Synthesis and biological activity evaluation of a novel pleuromutilin derivative 22-((2-methyl-1-(1H-pyrrole-2-carboxamido)propan-2-yl)thio)-deoxypleuromutilin
Source: Front Pharmacol. 2025 Sep 5;16:1657973. doi: 10.3389/fphar.2025.1657973 (PMC12447641; doi:10.3389/fphar.2025.1657973)

Supplementary Information

Synthesis and biological activity evaluation of a novel pleuromutilin derivative 22-((2-methyl-1-(1H-pyrrole-2-carboxamido)propan-2-yl)thio)-deoxypleuromutilin

Zhun Li, Danqian Ma, Shihong Li, Zhe Qin, Lixia Bai, Wenbo Ge, Xiao Xu, Jianyong Li^*^

Key Lab of New Animal Drug Project of Gansu Province, Key Lab of Veterinary Pharmaceutical Development of Ministry of Agriculture and Rural Affairs, Lanzhou Institute of Husbandry and Pharmaceutical Sciences of CAAS, Lanzhou, China

HR MS, ^1^H NMR and ^13^C NMR spectrum of compound **PDP**

HPLC spectra data for tested compound **PDP**

**HR MS, ^1^H NMR and ^13^C NMR spectrum of compound PDP**

**PDP#**


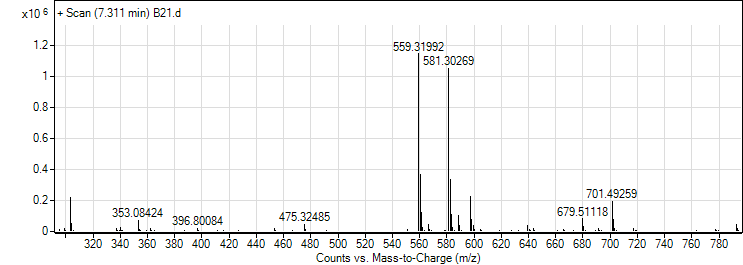

**HPLC spectra data for tested compound PDP**

The purity of compound **PDP** was determined by HPLC analysis (UV detection at 212 nm) confirming to be over 95%.


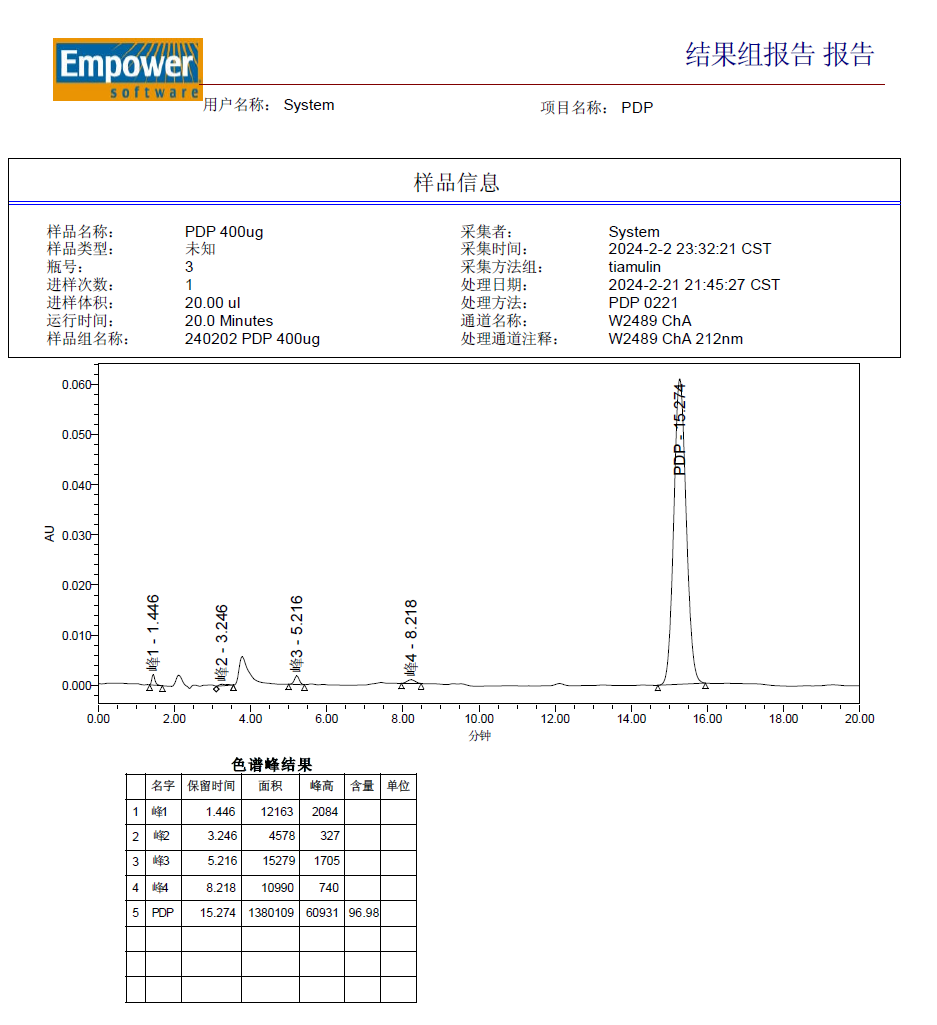

Supplement: Supplementary file 1 [file DataSheet1.docx]
